# An acceptor analogue of β-1,4-galactosyltransferase: Substrate, inhibitor, or both?

**DOI:** 10.1016/j.carres.2017.08.012

**Published:** 2017-10-10

**Authors:** Jingqian Jiang, Gerd K. Wagner

**Affiliations:** King's College London, Department of Chemistry, Faculty of Natural & Mathematical Sciences, Britannia House, 7 Trinity Street, London, SE1 1DB, UK

**Keywords:** Glycosyltransferase, Acceptor, Inhibitor, Substrate

## Abstract

Many glycosyltransferase inhibitors in the literature are structurally derived from the donor or acceptor substrate of the respective enzyme. A representative example is 2-naphthyl β-d-GlcNAc, a synthetic GlcNAc glycoside that has been reported as a galactosyltransferase inhibitor. This GlcNAc derivative is attractive as a chemical tool compound for biological and biochemical studies because of its reported potency as an inhibitor, and its short and straightforward synthesis from readily available starting materials. We report that in our hands, 2-naphthyl β-d-GlcNAc behaved, unexpectedly, as an acceptor substrate of the inverting β-1,4-galactosyltransferase (β-1,4-GalT) from bovine milk. This substrate activity has not previously been described. We found that 2-naphthyl β-d-GlcNAc can be an acceptor substrate both for recombinantly expressed β-1,4-GalT, and for a commercial batch of the same enzyme, and both in the presence and absence of bovine serum albumin (BSA). As expected for a full acceptor substrate, this substrate activity was time- and concentration-dependent. Additional experiments show that the observed inhibitor/substrate switch is facilitated by a phosphatase that is an essential component of our enzyme-coupled glycosyltransferase assay. These findings suggest that the behaviour of 2-naphthyl β-d-GlcNAc and related acceptor-based glycosyltransferase inhibitors is strongly dependent on the individual assay conditions. Our results therefore have important implications for the use of 2-naphthyl β-d-GlcNAc and related glycosides as tool compounds in glycobiology and glycobiochemistry.

## Highlights

• The known β-1,4-galactosyltransferase inhibitor GlcNAc β1-(2-naphthyl) can also behave as an acceptor substrate.
• This acceptor substrate activity is promoted by the presence of a phosphatase in the assay mixture.
• A kinetic model is proposed that reconciles the substrate and inhibitory activity of GlcNAc β1-(2-naphthyl).

## Graphical abstract

**Figure:**
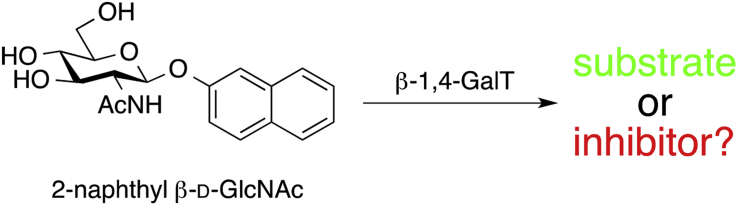

## 1 Introduction

Glycosyltransferases (GTs) are key enzymes for glycan biosynthesis, which catalyse the transfer of a sugar from a glycosyl donor, e.g. a sugar-nucleotide, to a suitable acceptor [1]. GTs are involved in many fundamental biological processes contingent on glycan epitopes, such as cell adhesion and cancer metastasis [2], [3]. GT inhibitors are therefore sought after as chemical tools for glycobiology and as potential lead compounds for drug discovery [4].

**Fig. 1:**
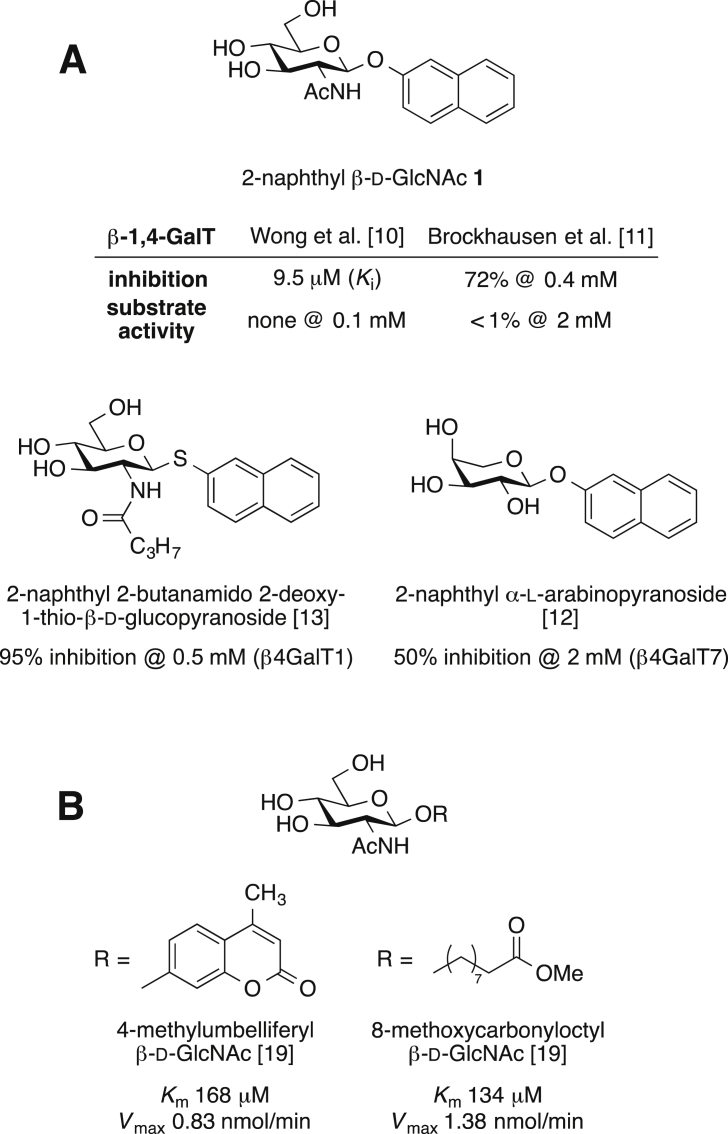
GalT acceptor analogues discussed in the text that have been reported as inhibitors (A) or substrates (B).

An important member of the GT family is the inverting galactosyltransferase β-1,4-GalT, which catalyses the transfer of d-galactose from a UDP-α-d-galactose (UDP-Gal) donor to *N*-acetylglucosamine (GlcNAc)-containing acceptors [5]. The resulting Galβ-1,4-GlcNAc units are found in many different glycoconjugates, and an isoform of β-1,4-GalT has recently been identified as a major control point for glycan branching in N-linked glycosylation [6]. β-1,4-GalT has been used extensively as a model target for the development of GT inhibitors, including substrate analogues based on either donor [7], [8] or acceptor [9]. Amongst the most potent acceptor-based β-1,4-GalT inhibitors are hydrophobic GlcNAc glycosides such as 2-naphthyl β-d-GlcNAc **1** (Fig. 1), for which low micromolar inhibitory activity has been reported by Wong and co-workers [10]. Due to the presence of the 4-OH group, in principle, **1** could still behave as a substrate towards β-1,4-GalT. However, two independent studies have described **1** as practically devoid of acceptor substrate activity [10], [11]. Similar profiles towards GalTs have also been reported for other 2-naphthyl glycosides (Fig. 1) [12], [13].

For an ongoing programme on the discovery of new GT inhibitor chemotypes, we required an established β-1,4-GalT inhibitor as a positive control. In the absence of a commercially available β-1,4-GalT inhibitor, we selected 2-naphthyl β-d-GlcNAc **1** because of its potency [10] and simple, two-step synthesis [14]. The only structural difference between **1** and the natural β-1,4-GalT acceptor substrate GlcNAc is the presence of the naphthyl aglycon. It has been reported that the presence of this substituent is sufficient to turn **1** from a β-1,4-GalT substrate into an inhibitor [11]. The inhibitory activity of **1** and several related GlcNAc derivatives has been attributed in particular to the lipophilicity of this substituent in the anomeric position [11].

Herein, we report that in our hands, **1** acted not as an inhibitor but, unexpectedly, as an acceptor substrate of β-1,4-GalT. These results are in contrast to the previously reported inhibitory activity of this GlcNAc derivative towards β-1,4-GalT [10], [11]. This substrate activity of **1** was observed both with a batch of bovine β-1,4-GalT recombinantly expressed in our own laboratory, and a commercial batch of the same enzyme, and both in the presence and absence of bovine serum albumin (BSA). Further experiments revealed that this unexpected substrate activity is promoted by a phosphatase, which is an essential component of our GT assay mixture. Galactosylation of **1** was observed by LC/MS both in the presence and absence of phosphatase. This profile suggests that this secondary enzyme reveals the latent acceptor substrate activity of **1** by altering the equilibrium position of several interconnected enzymatic reactions in the assay. This observation has important implications for the use of **1** and related glycosides as a tool compounds in glycobiology.

## 2 Results

**Scheme 1:**
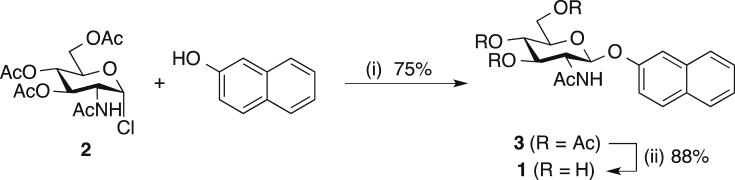
Synthesis of 2-naphthyl β-d-GlcNAc **1**[14]. *Reagents and conditions*: (i) tetrabutylammonium bromide, 1N NaOH, DCM, rt, 2 h; (b) NaOMe, MeOH/toluene (1:1), rt, 0.5 h.

2-Naphthyl β-d-GlcNAc **1** was synthesized in two steps according to the literature procedure [14]. Nucleophilic substitution of commercial 2-acetamido-3,4,6-tri-*O*-acetyl-2-deoxy-α-d-glucopyranosyl chloride **2** with 2-naphthol gave the fully acetylated derivative **3**, which was deprotected with 0.05M sodium methoxide to afford the desired 1-(2-naphthyl) 2-acetamido-2-deoxy-β-d-glucopyranoside **1** (Scheme 1). Results from the analytical characterisation of **1** by ^1^H- and ^13^C- NMR and mass spectrometry were consistent with data reported in the literature [14]. In particular, the β-configuration at the anomeric position was unambiguously established based on the coupling constant between H-1 and H-2 (*J* = 8.4 Hz). This *J* value is indicative of the *trans* orientation of H-1 and H-2, and hence the β-configuration of the aglycon at the anomeric position of **1**.

Although **1** has been reported as a β-1,4-GalT inhibitor, this monosaccharide can, in principle, also behave as a substrate towards that enzyme. With sufficient quantities of compound **1** in hand, we therefore decided to first test the potential substrate activity of **1** towards bovine β-1,4-GalT. For these experiments, we used a recently developed biochemical glycosyltransferase assay [15], [16]. In this coupled assay, a phosphatase is used to quantitatively release two equivalents of inorganic phosphate from each molecule of UDP, the secondary product of the β-1,4-GalT reaction. The free phosphate is then quantified colorimetrically with a malachite green reagent [15], [16]. For the substrate experiments, we used **1** in place of the standard acceptor substrate GlcNAc. In initial experiments, we evaluated the effects of acceptor concentration and incubation time on β-1,4-GalT activity with either **1** or GlcNAc as the acceptor substrate. Experiments with either the standard acceptor substrate GlcNAc or the putative inhibitor **1** were conducted in parallel on the same microplate. To account for the potential non-specific hydrolysis of the UDP-Gal donor substrate, we also included relevant control wells on each microplate (no acceptor, but otherwise identical conditions). We also carried out separate control experiments to identify potential interference of **1** with the colorimetric readout of our assay (no enzyme, but otherwise identical conditions).

**Fig. 2:**
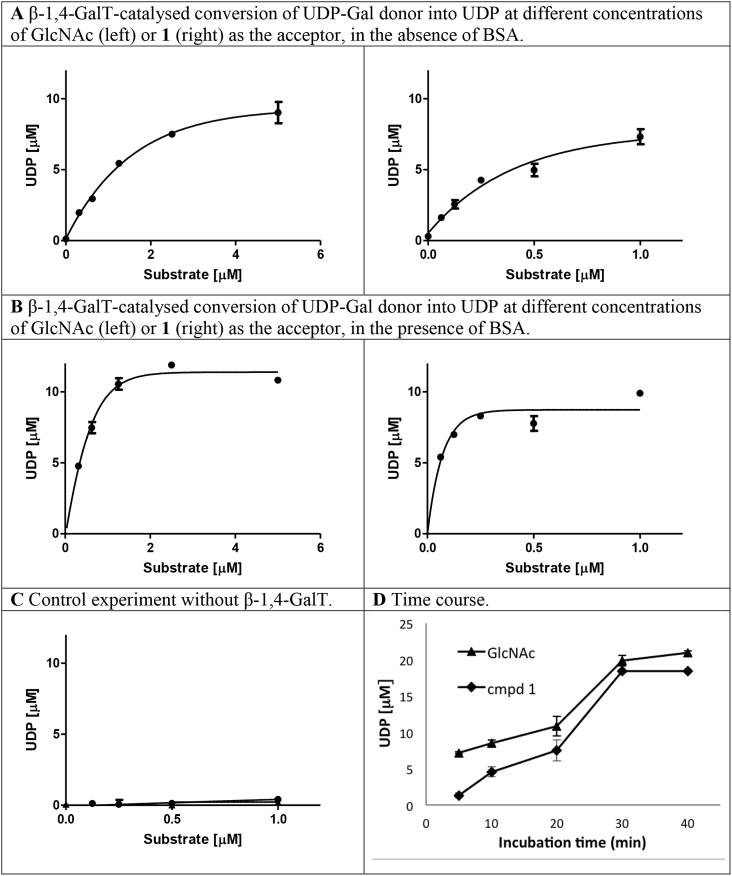
Acceptor substrate assays with GlcNAc or **1**, and recombinant β-1,4-GalT. *Conditions*: All experiments were carried out in triplicate. Bars indicate mean values ± S.D. **A** β-1,4-GalT, GlcNAc (0–5 mM) or **1** (0–1 mM), UDP-Gal donor (28 μM), MnCl_2_ (5 mM), chicken egg-white lysozyme (1 mg/mL), calf-intestinal phosphatase (10 U/mL), DMSO (10%) and buffer (13 mM HEPES, pH 7.0, 50 mM KCl) were incubated in a 96-well plate at 30 °C with shaking for 20 min. The reaction was stopped by the addition of malachite reagents, and the absorbance was recorded at 620 nm after 30 min. **B** Conditions as in **A** but with bovine serum albumin (1.25 mg/mL). **C** Conditions as in **A** with compound **1** (0–1 mM) as the substrate, but without β-1,4-GalT. **D** Conditions as in **A**, with GlcNAc (5 mM) or **1** (1 mM) as the acceptor, and with incubation times from 5 to 40 min.

Significant β-1,4-GalT activity was observed in both cases, whether GlcNAc or GlcNAc derivative **1** was present as potential acceptor substrate (Fig. 2A). The observed β-1,4-GalT activity was dependent on the concentration of the respective acceptor. GlcNAc derivative **1** itself does not interfere with the colorimetric readout of our assay, as evidenced by the lack of signal in the control experiment with increasing concentrations of **1** but without enzyme (Fig. 2C). Interestingly, from these experiments a slightly lower *K*_m_ value can be determined for GlcNAc derivative **1** than for GlcNAc (0.2 μM vs 1 μM), suggesting that **1** may actually be a better acceptor substrate than GlcNAc. To investigate the time dependency of the β-1,4-GalTs reaction under these conditions, we next incubated the reaction with a single concentration of GlcNAc (5 mM) or **1** (1 mM) for 5–40 min. A clear time-dependency of turnover was observed with both acceptors (Fig. 2D). With increasing incubation time, UDP formation increased in the presence of both GlcNAc and **1**, with a maximal turnover after 30 min. Taken together, these results suggest strongly that, just like its parent compound GlcNAc, GlcNAc derivative **1** is recognised as an acceptor substrate by bovine β-1,4-GalT.

In order to understand this discrepancy with previous literature reports [10], [11], we carried out additional experiments. In the main literature precedent, β-1,4-GalT from bovine milk was obtained commercially, and the assay was carried out in the presence of 12.5 mg/mL BSA [11]. While BSA is frequently added to assay mixtures to generically reduce protein adhesion to the reaction container, in the case of GTs, a direct influence of BSA on substrate specificity, as well as the kinetic parameters of the enzymatic reaction, has also been observed [17]. To assess the effect of BSA on the behaviour of GlcNAc derivative **1**, we repeated the substrate activity assay in the presence of BSA (12.5 mg/mL). Assay mixtures were incubated for 20 min and absorbance was recorded at 620 nm. When the formation of UDP was plotted over the concentration of acceptor (GlcNAc or **1**), once again very similar reaction profiles were observed for both GlcNAc and GlcNAc derivative **1** (Fig. 2B). The absence of BSA from our initial experiments could therefore be ruled out as a potential explanation for the observed discrepancy with the previous literature results. Interestingly, the turnover rates for both GlcNAc and **1** were higher in the presence of BSA than in its absence (Fig. 2B). This effect of BSA is in keeping with observations for another glycosyltransferase [17].

While our initial experiments were all carried out with a batch of bovine β-1,4-GalT recombinantly expressed in our own laboratory, previously reported results for **1** were obtained with a commercially available enzyme [11]. The information provided by the supplier about this β-1,4-GalT is limited, and it was therefore not certain if it was identical to our own recombinant batch. We therefore decided to repeat our experiments with a batch of commercial β-1,4-GalT from the same supplier. First, we determined the activity of the commercial β-1,4-GalT as 12.7 mU/mL (ESI). Then, we repeated the substrate assays with either GlcNAc or **1** as acceptor, in the absence or presence of BSA. The results were practically identical to those obtained previously with recombinantly expressed enzyme. GlcNAc derivative **1** once again acted as a substrate towards β-1,4-GalT, both in the absence and presence of BSA (Fig. S1). In the presence of BSA, turnover rates increased approximately 2-fold, for both GlcNAc and **1**. The *K*_m_ value of each substrate also increased, by approximately 4-fold (GlcNAc: 0.4 μM without BSA, 1.5 μM with BSA; **1**: 0.08 μM without BSA, 0.3 μM with BSA). Most importantly, both trends (increased turnover, increased *K*_m_) are practically identical for both GlcNAc and GlcNAc derivative **1**. This provides further evidence that both monosaccharides can serve, in an identical manner, as acceptor substrates for β-1,4-GalT.

Our standard assay does not detect the primary product of the GalT reaction, in contrast to the radiochemical GT assay used in the original reports of **1** as a β-1,4-GalT inhibitor [10], [11]. In order to directly detect the putative galactosylation product of **1**, we therefore carried out LC/MS experiments. **1** (500 μΜ), recombinant β-1,4-GalT (200 μL) and UDP-Gal (500 μΜ) were incubated for 1 h at 30 °C in HEPES buffer (pH 7.0, 50 mM KCl). Analysis of the reaction by LC/MS showed the appearance of a new peak in the chromatogram, at a slightly shorter retention time than the peak corresponding to **1** (Fig. S2). While the separation of the two peaks is not complete, the mass spectra confirmed the formation of a species with a molecular ion of 532, which corresponds to the sodium adduct of mono-galactosylated **1**, Gal-**1**. This result provides direct evidence that **1** can indeed act as an acceptor substrate for β-1,4-GalT. The formation of Gal-**1** was strongly dependent on the amount of β-1,4-GalT present, and significant levels of the galactosylated species were only observed at the highest enzyme concentration tested (Fig. S3, **A**-**C**). Interestingly, however, if phosphatase (20 μL) was present in the reaction mixture, similar levels of Gal-**1** were observed even at a lower β-1,4-GalT concentration (Fig. S3, **D**). The conditions used for this last experiment are identical to the ones used in our standard assay protocol. These results therefore suggest that the enzymatic profiles which we observed in our biochemical assays were indeed due to the behaviour of **1** as an acceptor substrate of β-1,4-GalT.

## 3 Discussion

Building on the seminal work by Lemieux [18], Palcic has previously shown that GlcNAc glycosides with a hydrophobic aglycon, such as 8-methoxycarbonyloctyl GlcNAc and 4-methylumbelliferyl GlcNAc (Fig. 1), are highly effective acceptor substrates for β-1,4-GalT [19]. Intriguingly, the closely related GlcNAc derivative **1** has been reported in two separate studies as an inhibitor, but not a substrate, of β-1,4-GalT [10], [11]. In our hands, however, **1** did behave as an acceptor substrate towards this enzyme, with a similar profile as the natural GlcNAc acceptor. This discrepancy is not due to a case of “mistaken identity” of **1**. Spectroscopic characterisation of our batch of **1** and careful comparison with literature data [14] confirms that the compound used in this study is identical to the one that had been reported previously.

Our results suggest that the behaviour of **1** towards β-1,4-GalT is strongly dependent on the assay conditions. In the literature precedent, GlcNAc derivative **1** and its analogues were tested in a radiochemical GT assay, which directly detects the galactosylated reaction product [11]. The enzyme-coupled assay used in the present study requires a phosphatase, in conjunction with a colorimetric read-out, to measure the formation of UDP, the secondary product of the β-1,4-GalT reaction [16]. We have eliminated potential false positives that would specifically affect our assay protocol, e.g. from non-specific hydrolysis of UDP, by including the requisite control experiments. We have also investigated the role of specific assay components used in the original assay, such as BSA, and the source of the β-1,4-GalT. We found that these parameters do not fundamentally alter the outcome of the reaction. These results demonstrate that despite the different assay formats, the results from the radiochemical assay used in the previous study, and from our own colorimetric assay are, in principle, comparable.

Despite the seeming contradiction, our findings are not irreconcilable with the previous results. We have observed substrate activity for **1** both in the presence and absence of phosphatase. This suggests that while the presence of the phosphatase in our assay facilitates the utilisation of **1** as a β-1,4-GalT acceptor substrate, it is not essential. The addition of phosphatase or NDP-cycling enzymes is a well-established approach in chemo-enzymatic applications of GTs, to drive the GT reaction to completion and to improve the efficiency of the overall synthetic process [20]. It is likely that the presence of the phosphatase in our assay affects the equilibrium between different species along the β-1,4-GalT kinetic pathway in a similar fashion.

**Fig. 3:**
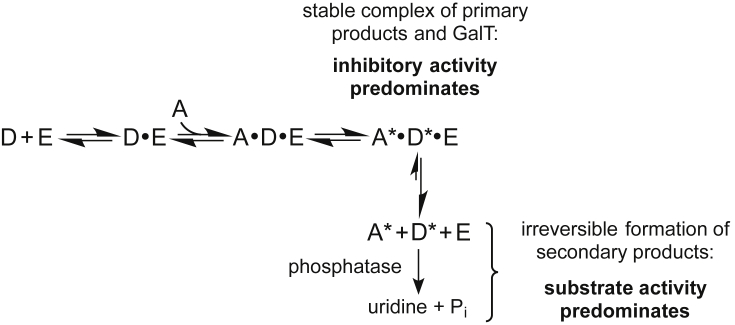
Hypothetical model for the dual activity of **1** as either acceptor substrate or inhibitor along the β-1,4-GalT kinetic pathway (D: donor sugar-nucleotide, E: enzyme, A: acceptor, A^∗^: galactosylated acceptor, D^∗^: nucleoside diphosphate).

We propose the following model, which is consistent with both our own data and the previous findings (Fig. 3): because of its close structural similarity to the natural acceptor GlcNAc, **1** can indeed be recognised as an acceptor substrate by β-1,4-GalT, which converts it into the corresponding disaccharide Gal-**1**. This disaccharide may form a stable, non-covalent complex with the enzyme, acting effectively as a feedback inhibitor. This would be consistent with the previous finding that a hydrophobic aglycone such as 2-napthyl is crucial for strong substrate binding in this series [11]. This inhibitory effect can be overcome either by increasing the concentration of β-1,4-GalT, or by including phosphatase in the assay mixture. The irreversible, phosphatase-catalysed hydrolysis of UDP will shift the overall equilibrium towards the reaction products, thus revealing the latent substrate activity of **1** even at low β-1,4-GalT concentrations (Fig. 3). It has to be emphasised that at present, this model is hypothetical. Its definitive test will require the synthesis and testing of the Gal-**1** disaccharide. While disaccharide product inhibition of glycosyltransferases is perhaps less common than inhibition by the NDP reaction product, there is precedent for the inhibition of β-1,4-GalT by disaccharide acceptor analogues [21].

## 4 Conclusion

In conclusion, we have found that 2-naphthyl β-d-GlcNAc **1** can behave as an acceptor substrate towards β-1,4-GalTs from bovine milk. This substrate activity has not previously been described. Its discovery was facilitated by the presence of a phosphatase in our enzyme-coupled GT assay, which affects the reaction equilibria along the β-1,4-GalT kinetic pathway. The finding that **1** can behave as a β-1,4-GalT substrate has implications both for the design and the application of acceptor-based GT inhibitors: in principle, **1** and related GT inhibitors are attractive chemical tools to study glycosylation in cells. Our results suggest that such applications in cells may be complicated by the presence of phosphatases, which will very likely modulate the inhibitor/substrate behaviour of such acceptor analogues. Following conversion of **1** into the corresponding disaccharide, it may be this disaccharide that acts as the actual inhibitory species through a feedback inhibition mechanism. In this case, the substrate activity of **1** would be a prerequisite for its inhibitory activity. Given the considerable number of acceptor-based GT inhibitors in the literature, these questions warrant further investigation. The present study provides a starting point for such investigations.

## 5 Experimental section

**General.** All reagents were obtained commercially and used as received unless stated otherwise. GlcNAc derivative **1** was synthesized as previously reported and characterised by ^1^H- and ^13^C-NMR spectroscopy and mass spectrometry [14]. Thin-layer chromatography (TLC) was performed on pre-coated aluminium plates (Silica Gel 60 F_254_, Merck) and compounds were visualised by exposure to UV light (254 and 280 nm). Preparative chromatography was carried out on silica gel 60 (pore size 60 Å, 230–400 mesh, Sigma-Aldrich) at normal pressure. NMR spectra were recorded at 298K on a Bruker Avance DRX 400 spectrometer (400 MHz for ^1^H, 100 MHz for ^13^C). Chemical shifts (*δ*) are reported in ppm (parts per million). Coupling constants (*J*) are reported in Hz.

**2-Naphthyl 3,4,6-tri-*O*-acetyl 2-acetamido-2-deoxy-*β*-****d****-glucopyranoside (3)** [14]. To an aqueous solution of sodium hydroxide (1M, 2 mL), a solution of 3,4,6-tri-*O*-acetyl-2-deoxy-α-D-glucopyranosyl chloride **2** (400 mg, 1 equiv.), 2-naphthol (319 mg, 2 equiv.) and tetra-*n*-butylammonium bromide (354 mg, 1 equiv.) in methylene chloride (2 mL) was added. The resulting two-phase system was stirred for 2 h at rt. The mixture was diluted with ethyl acetate and the organic phase washed sequentially with an aqueous solution of 1M sodium hydroxide and water, then dried. The organic extract was then filtered, and the filtrate concentrated to yield a crude solid which was purified by chromatography (DCM/MeOH 20:1), to yield 389 mg of a white powder (75%). ^1^H-NMR (400 MHz, CDCl_3_): *δ* 7.81 (d, 2H, *J* = 9.0 Hz, H-4 and H-9 of naphthyl), 7.77 (d, 1H, *J* = 8.0 Hz, H-6 of naphthyl), 7.47 (m, 1H, H-7 of naphthyl), 7.44 (d, 1H, *J* = 2.5 Hz, H-1 of naphthyl), 7.38 (m, 2H, H-8, H-5 of naphthyl), 7.22 (dd, 1H, *J* = 2.5, 9.0 Hz, H-3 of naphthyl), 5.46 (d, 1H, *J* = 8.0 Hz, H-1), 5.39 (dd, 1H, *J* = 10.5, 10.5 Hz, H-3), 5.11 (dd, 1H, *J* = 10.5, 10.5 Hz, H-4), 4.36 (dd, 1H, *J* = 5.5, 12.5 Hz, H-6), 4.16 (M, 2H, H-2 and H-6), 4.11 (m, 1H, H-5), 2.07, 2.06, 2.04 (3 × s, 9H, acetyl), 1.96 (s, 3H, acetamido). ^13^C-NMR (100 MHz, CDCl_3_): *δ* 20.8, 20.9, 20.9, 23.3, 59.0, 61.9, 71.0, 74.6, 78.0, 82.3, 121.0, 126.1, 126.5, 127.9, 128.0, 130.3, 137.4, 128.5, 148.1, 156.9, 170.4, 171.4, 171.8, 172.5.

**1-(2-Naphthyl) 2-acetamido-2-deoxy-β-D-glucopyranoside (1)** [14]. To a solution of **3** (200 mg) in methanol-toluene (1:1) was added a catalytic amount of 0.5M sodium methoxide in methanol. The reaction mixture was stirred at room temperature for 0.5 h and the progress of the reaction was monitored by TLC (DCM/MeOH 4:1). Upon completion of the reaction, the organic solution was concentrated, and the residue was purified by chromatography (DCM/MeOH 4:1), to yield 92 mg of a white powder (88%). ^1^H-NMR (400 MHz, MeOD): *δ* 7.76 (m, 3H, H-4, H-9, H-6 of naphthyl), 7.42 (m, 2H, H-7, H-1 of naphthyl), 7.34 (m, 1H, H-8 of naphthyl), 7.18 (dd, 1H, *J* = 2.4, 9.2 Hz, H-3 of naphthyl), 5.19 (d, 1H, *J* = 8.4 Hz, H-1), 4.00 (dd, 1H, *J* = 8.4, 10.4Hz, H-2), 3.98 (dd, 1H, *J* = 2.4, 12Hz, H-6), 3.77 (1H, dd, *J* = 5.6, 12Hz, H-6), 3.63 (dd, 1H, *J* = 8.4, 10Hz, H-3), 3.58 (m, 1H, H-5), 3.48 (dd, 1H, *J* = 8.8, 9.6Hz, H-4), 1.99 (s, 3H, NHCH_3_). ^13^C-NMR (100 MHz, MeOD): δ 23.0, 57.4, 62.6, 71.9, 75.9, 78.4, 101.0, 111.9, 119.8, 125.3, 127.4, 128.2, 128.6, 130.4, 131.3, 135.9, 156.9, 174.0. m/z (ESI) 371.1199 [M + H + Na]^2+^, C_18_H_22_NNaO_6_ requires 371.1345.

**Enzymology.** The plasmid for β-1,4-GalT from bovine milk was a generous gift from Dr Christelle Breton (Grenoble). Bovine β-1,4-GalT was either expressed in our own laboratory as previously reported [7] or obtained commercially (Sigma). For the biochemical assays, we used a recently reported colorimetric protocol [16]. All assays were carried out in Nunc clear, flat-bottom 96-well polystyrene microplates. Assay wells typically contained MnCl_2_, calf-intestinal phosphatase (CIP), chicken egg-white lysozyme (CEL), UDP-Gal donor and either GlcNAc or **1** as acceptor (for details of the assay protocol see ESI). To quantify the concentration of inorganic phosphate (P_i_), malachite green reagents were added, and the absorbance was recorded at 620 nm on a BMG Labtech POLARstar Optima multiplate reader.

**Data collection and analysis.** A calibration curve (0–12.5 μM UDP, corresponding to 0–25 μM P_i_) was constructed for each microplate by linear regression. The calibration curve was used to convert absorbance measurements at 620 nm in sample and control wells to [UDP] (μM). For each sample and control well, a corresponding background well (containing identical components but no acceptor) was included, to account for non-specific hydrolysis of donor. Corrected absorbance values for each well were obtained by subtracting the corresponding background reading from the absorbance of the respective sample or control well. The calculated concentration of UDP was plotted against concentration of acceptor (for substrate assay or control assay) or incubation time (for time-dependent assays). Averages and standard deviations were calculated in Microsoft Excel.

## 6 LC/MS experiments

Standard assay mixtures for investigating the acceptor properties of compound **1** towards β-1,4-GalT contained UDP-Gal (500 μΜ), compound **1** (500 μΜ), β-1,4-GalT (20, 50 or 200 μL, Fig. S3), calf intestinal phosphatase (20 μL, assay D only, Fig. S3), 13 mM HEPES buffer (pH 7.0, 50 mM KCl). Mixtures were incubated for 1 h at 30 °C in a water bath. Reactions were stopped by the addition of the same volume of methanol. The mixtures were centrifuged for 15 min at 1000 rpm. The supernatants were used for LC/MS analysis directly. LC/MS analysis was carried out on an HPLC reverse phase column (Agilent Eclipse XDB-C8 4.6 × 150 mm) with water (0.1% formic acid) against methanol as the mobile phase. The gradient is shown in Table 1. The HPLC was coupled to an Advion Compact Mass Spectrometer (CMS) for mass detection.
