# Supplementary material for: An acceptor analogue of β-1,4-galactosyltransferase: Substrate, inhibitor, or both?
Source: Carbohydr Res. 2017 Oct 10;450:54–9. doi: 10.1016/j.carres.2017.08.012 (PMC5636615; doi:10.1016/j.carres.2017.08.012)
Supplement: Online data [file mmc1.docx]

**SUPPLEMENTARY INFORMATION**

**An acceptor analogue of β-1,4-galactosyltransferase: substrate, inhibitor, or both?**

Jingqian Jiang & Gerd K. Wagner*

King’s College London, Department of Chemistry, Faculty of Natural & Mathematical Sciences, Britannia House, 7 Trinity Street, London, SE1 1DB, UK.

Phone: +44 (0)20 7848 1926

e-mail: [gerd.wagner@kcl.ac.uk](mailto:gerd.wagner@kcl.ac.uk)

CONTENT

1. NMR spectra of **1**
2. Additional figures
3. Activity of commercial β-1,4-GalT
4. Enzyme assay protocol
5. HPLC elution gradient for LC/MS experiments
6. **^1^H- and ^13^C-NMR spectra of 2-naphthyl β-d-GlcNAc 1**

**(2) Additional figures**

**Fig. S1** Acceptor substrate assays with commercial β-1,4-GalT in the absence or presence of BSA.^a^

(a) β-1,4-GalT-catalysed conversion of UDP-Gal donor at different concentrations of GlcNAc (left) or **1** (right) as the acceptor, in the absence of BSA.

(b) β-1,4-GalT-catalysed conversion of UDP-Gal donor at different concentrations of GlcNAc (left) or **1** (right) as the acceptor, in the presence of BSA.

^a^*Conditions*: (a) β-1,4-GalT, GlcNAc (0-5 mM) or **1** (0-1 mM), UDP-Gal donor (28 μM), MnCl_2_ (5 mM), chicken egg-white lysozyme (1 mg/mL), calf-intestinal phosphatase (10 U/mL), DMSO (10%) and buffer (13 mM HEPES, pH 7.0, 50 mM KCl) were incubated in a 96-well plate at 30 ^o^C with shaking for 20 min. The reaction was stopped by the addition of malachite reagents, and the absorbance was recorded at 620 nm after 30 min. (b) Conditions as in (a) but with addition of bovine serum albumin (1.25 mg/mL). All experiments were carried out in triplicate. Bars indicate mean values ± S.D.

**Fig. S2** LC/MS analysis of the β-1,4-GalT-catalysed reaction with **1** as acceptor. **A:** Full chromatogram showing the peaks for **1** (t_R_ 35.6 min, m/z 370 [M+Na]^+^) and the galactosylated product **1**-Gal (t_R_ 34.9 min, m/z 532 [M + Na]^+^). **B**: Expansion of **A**. **C**: Mass spectrum for the peak at 34.9 min. **D**: Mass spectrum for the peak at 35.6 min.^a^

^a^*Conditions*: UDP-Gal (500 μM), compound **1** (500 μM), recombinant β-1,4-GalT (200 μL) and buffer (13 mM HEPES, pH = 7.0, 50 mM KCl) were incubated in plastic vials for 1h at 30 ^o^C in a water bath. Reactions were stopped by the addition of the same volume of methanol. The mixtures were centrifuged for 15 min at 1000 rpm. The supernatants were used for LC/MS analysis directly. LC/MS analysis was carried out on a reverse phase column (Agilent Eclipse XDB-C8, 4.6×150 mm), with water (0.1 % formic acid) against methanol as the mobile phase. The gradient is shown in Table **1**.

**Fig. S3** Effect of β-1,4-GalT concentration and presence of phosphatase on the formation of galactosylated **1**. **A:** β-1,4-GalT (20 μL). **B:** β-1,4-GalT (50 μL). **C:** β-1,4-GalT (200 μL). **D:** β-1,4-GalT (50 μL) and phosphatase (20 μL).^a^


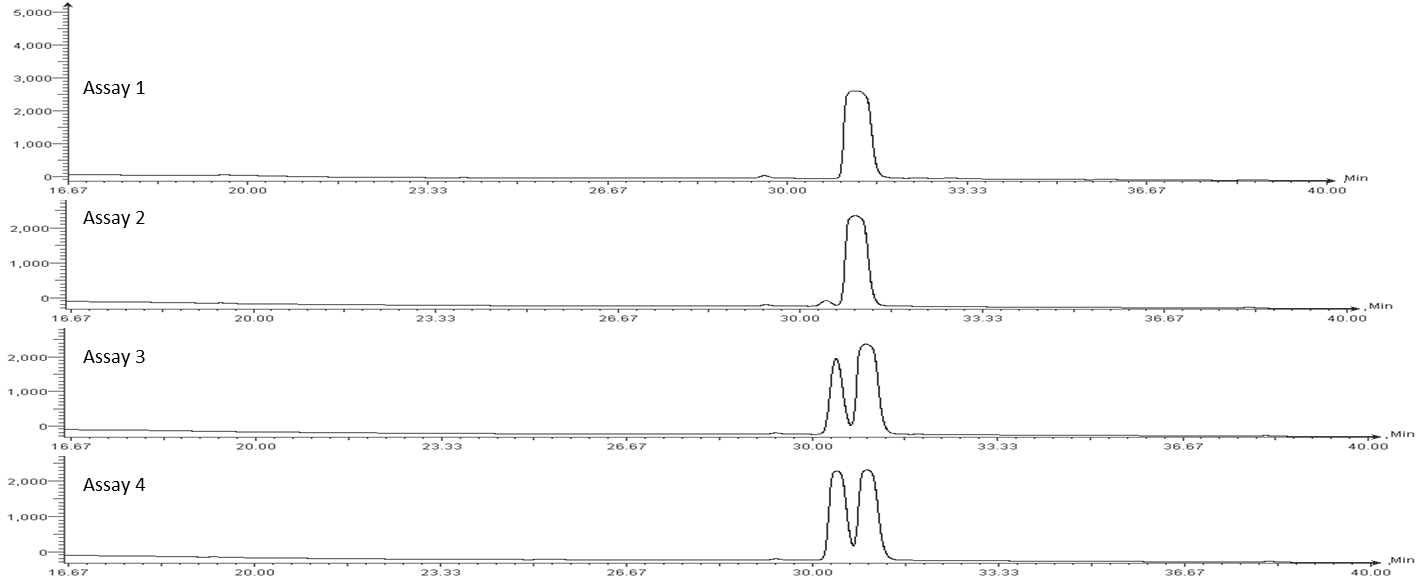


**1**

**1**-Gal

**D** β-1,4-GalT (50 μL) + phosphatase (20 μL)

**C** β-1,4-GalT (200 μL)

**B** β-1,4-GalT (50 μL)

**A** β-1,4-GalT (20 μL)

^a^*Conditions*: UDP-Gal (500 μM), compound **1** (500 μM), recombinant β-1,4-GalT (20, 50 or 200 μL), phosphatase (20 μL utilized in assay D) and buffer (13 mM HEPES, pH 7.0, 50 mM KCl) were incubated in plastic vials for 1h at 30 ^o^C in a water bath. Reactions were stopped by the addition of the same volume of methanol. The mixtures were centrifuged for 15 min at 1000 rpm. The supernatants were used for LC/MS analysis directly. LC/MS analysis was carried out on a reverse phase column (agilent eclipse XDB-C8 4.6×150 mm), with water (0.1 % formic acid) against methanol as the mobile phase. The gradient is shown in Table **1**.

**(3) Activity assay of commercial β-1,4-GalT^a^**


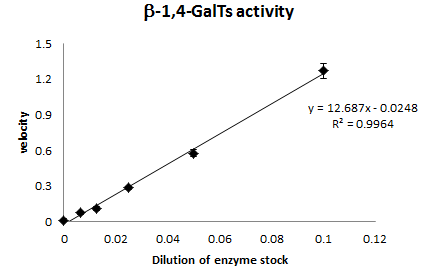


^a^*Conditions*: β-1,4-GalTs (diluted to the required concentration), GlcNAc acceptor (5 mM), UDP-Gal donor (28 μM), MnCl_2_ (5 mM), chicken egg-white lysozyme (1 mg/mL), calf-intestinal alkaline phosphate (10 U/mL), DMSO (10%) and buffer (13 mM HEPES, pH 7.0, 50 mM KCl) were incubated in a 96-well plate at 30 ^o^C with shaking for 20 min. The reaction was stopped by the addition of malachite reagents, and the absorbance was recorded at 620 nm after 30 min. All concentrations are final concentrations. Bars indicate mean values ± S.D. of triplicate experiments.

**(4) Enzyme assay protocol**

The general assay protocol followed the previously published procedure (Tedaldi et al. *MedChemComm* 2014; **5**: 1193-201). First, the components (including MnCl_2_, CIP, CEL, buffer, enzyme, DMSO) were prepared in the master-mix and added into each well. GlcNAc was added into the wells and was thereafter replaced by the buffer for background and **1** wells. Similarly, the same procedure was repeated for **1**. To start the enzyme reaction, UDP-Gal was added in 15 μL aliquots of the respective stock solution. On each plate, a UDP calibration curve (0-12.5μM) was included. Wells for the calibration curve included all components of the standard reaction except for the acceptor and compound **1**. UDP was used as the starter instead of UDP-Gal. The reactions were incubated at 30 °C for 20 min, or for the requisite time in the time-dependency experiment. Reactions were stopped by consecutively adding malachite reagents A & B (30 μL each). The colour was allowed to develop for 30 min at 30 ^o^C, and the absorbance was measured at 620 nm.

**Master-mix preparation**

| Component | [stock] | [well] | Volume (μL) |
| --- | --- | --- | --- |
| MnCl_2_ | 50 mM | 5mM | 15 |
| CIP | 10,000 U/mL | 10 U/mL | 15 |
| CEL | 10mg/mL | 1mg/mL | 15 |
| Buffer | 13mM HEPES,  50 mM KCl, PH 7.0 | 13mM HEPES,  50 mM KCl, PH 7.0 | 30 |
| DMSO | 100% | 10% | 15 |
| Enzyme | 2.7 mU | 0.27 mU | 15 |
| Total |  |  | 105 |

**Well-map for substrate activity assays:**

| 1 | 2 | 3 | 4 | 5 | 6 | 7 | 8 | 9 | 10 |
| --- | --- | --- | --- | --- | --- | --- | --- | --- | --- |
| Calibration curve | | Background  (no acceptor) | | Assay A (GlcNAc)  mM | | | Assay B (**1**)  mM | | |
| 12.5 | 12.5 |  |  | 5 | 5 | 5 | 1 | 1 | 1 |
| 6.25 | 6.25 |  |  | 2.5 | 2.5 | 2.5 | 0.5 | 0.5 | 0.5 |
| 3.125 | 3.125 |  |  | 1.25 | 1.25 | 1.25 | 0.25 | 0.25 | 0.25 |
| 1.5625 | 1.5625 |  |  | 0.625 | 0.625 | 0.625 | 0.125 | 0.125 | 0.125 |
| 0.78 | 0.78 |  |  | 0.3125 | 0.3125 | 0.3125 | 0.0625 | 0.0625 | 0.0625 |
| 0 | 0 |  |  | 0 | 0 | 0 | 0 | 0 | 0 |

**Well-map for enzyme activity assays:**

| 1 | 2 | 3 | 4 | 5 | 6 | 7 | 8 | 9 | | 10 |
| --- | --- | --- | --- | --- | --- | --- | --- | --- | --- | --- |
| Calibration curve | | Background  (no acceptor) | | Assay A  Enzyme (dilution as a fraction of 1) | | Background  (no acceptor) | | Assay B  Enzyme (dilution as a fraction of 1) | | |
| 12.5 | 12.5 |  |  | 0.1 | 0.1 |  |  | 0.1 | 0.1 | |
| 6.25 | 6.25 |  |  | 0.05 | 0.05 |  |  | 0.05 | 0.05 | |
| 3.125 | 3.125 |  |  | 0.025 | 0.025 |  |  | 0.025 | 0.025 | |
| 1.5625 | 1.5625 |  |  | 0.0125 | 0.0125 |  |  | 0.0125 | 0.0125 | |
| 0.78 | 0.78 |  |  | 0.0063 | 0.0063 |  |  | 0.0063 | 0.0063 | |
| 0 | 0 |  |  | 0 | 0 |  |  | 0 | 0 | |

**(5) HPLC elution gradient for LC/MS experiments to separate 1 and Gal-1.**

| Gradient step | Time  (min) | Water/formic acid (0.1%)  (%) | Methanol  (%) |
| --- | --- | --- | --- |
| 1 | 1 | 90 | 10 |
| 2 | 10 | 90 to 70 | 10 to 30 |
| 3 | 20 | 70 to 50 | 30 to 50 |
| 4 | 30 | 50 to 30 | 50 to 70 |
| 5 | 40 | 30 to 20 | 70 to 80 |
| 6 | 70 | 20 | 80 |
| 7 | 80 | 20 to 90 | 80 to 10 |
| 8 | 85 | 90 | 10 |
